# Oncolytic Tanapoxvirus Recombinants Expressing Flagellin C or Mouse Interleukin-2 Are Capable of Regressing Human Triple-Negative Breast Cancer Xenografts in Immuno-Competent BALB/c Nude Mice

**DOI:** 10.3390/pathogens13050402

**Published:** 2024-05-13

**Authors:** Michael L. Monaco, Grace A. Filpi, Steven L. Kohler, Robert Eversole, Omer A. Idris, Karim Essani

**Affiliations:** Laboratory of Virology, Department of Biological Sciences, Western Michigan University, Kalamazoo, MI 49008, USA; michael.l.monaco@wmich.edu (M.L.M.); grace.a.filpi@wmich.edu (G.A.F.); steve.kohler@wmich.edu (S.L.K.); rob.eversole@wmich.edu (R.E.); omer.a.idris@wmich.edu (O.A.I.)

**Keywords:** Tanapoxvirus, oncolytic virus, triple-negative breast cancer, flagellin C, interleukin-2, immune reconstitution, anti-tumor response

## Abstract

Triple-negative breast cancer (TNBC) in humans is the most aggressive and deadly form of BC. Although TNBCs are about 15 percent of the total number of BC cases, they are associated with the highest mortalities. Current treatment options are limited, and most modalities are toxic and have not increased the 5-year survival rates of TNBC. Many oncolytic viruses are emerging as potential therapies for TNBC. In this study, two Tanapoxvirus (TPV) recombinants, one expressing FliC and the other expressing mouse interleukin-2 (mIL-2), were assessed for their efficacy in an immuno-competent xenograft mouse model. MDA-MB-231 tumors were planted in BALB/c nude mice, treated, made immuno-competent via adoptive transfer of splenocytes from healthy BALB/c donors, and then monitored for 40 days. TPV/Δ2L/66R/FliC and TPV/Δ66R/mIL-2 demonstrated significant tumor reduction (*p* = 0.01602 and *p* = 0.03890, respectively) compared to the reconstituted control (RC), whereas *wt*TPV did not. Pathological analyses of treated tumors revealed cells consistent with lymphocyte and plasma cell morphology in reconstituted mice treated with TPV recombinants. Anti-viral plaque reduction assays conducted using harvested serum from treated animals indicated the presence of anti-TPV antibodies in mice reconstituted and treated with TPV that were missing from immune-deficient nude mice, including those exposed to TPV and of statistically equivalent serum concentrations to normal BALB/c mice immunized against TPV. The results suggest immuno-deficient BALB/c nude mice can become immuno-competent via adoptive transfer of splenocytes from genetically identical donors and allow for testing of tumor xenografts in a competent model system. The TPV recombinants tested should be further studied for the potential treatment of human TNBC.

## 1. Introduction

Triple-negative breast cancer (TNBC) is the most serious form of BC in terms of survival, metastatic potential, and overall prognosis [1,2,3,4]. TNBC is diagnosed in approximately 15% of all BC cases yet causes the most deaths among all types of BC. Due to the lack of diagnostic biomarkers (estrogen, progesterone, and human epidermal growth factor receptor 2) that usually exist in some combination in other BC types, there are no targeted molecular therapies available for the treatment of TNBC. Instead, the standard of care for TNBC relies heavily upon chemotherapies, radiation, and surgical resection. Although TNBCs tend to respond favorably to chemotherapy, chemotherapeutics work via the 99% rule. There is a small population of cells that have escaped after each round of therapy, which allows the cancer cells to further divide and mutate, become resistant to, and ultimately survive these drugs. As a result, TNBC has the highest rate of tumor recurrence of any BC, and even worse, if TNBC metastasizes after a recurrence, the survival rate is essentially 0% [5].

Oncolytic viruses (OVs) are becoming a more prevalent alternative to standard cancer therapies because the natural world has provided platforms that can circumvent some of the obstacles that cancer cells provide to targeted molecular therapies (mutation/deletion of binding target). Paired with genetic engineering, viruses that would typically cause disease can be altered to become safe for administration while also recruiting the immune system to arrive at the tumor location and hopefully begin to detect antigens that previously were unavailable to adaptive immune responses. One of these OV platforms, Tanapoxvirus (TPV), has been engineered previously to express immuno-modulatory molecules such as mIL-2 and CCL2 [6,7,8] or pathogen-associated molecular patterns (PAMPs) like flagellin C from *Salmonella typhimurium* (FliC) [9] while deleting viral thymidine kinase (66R) and viral tumor necrosis factor-binding protein (2L) to keep viral replication to tumor cells and potentially increase anti-tumor immune responses. Previous studies in immuno-deficient (IDt) animal models have shown significant tumor responses compared to saline control (mock) with numerous TPV recombinants [6,7,8,9] across multiple cancer indications. 

A major obstacle to the development of TPV is finding a suitable animal model that can bear a study tumor while also maintaining immunocompetence (ICt). Most OVs have either the capability to infect mammalian model species’ tumors as well as humans or have analogous viruses that can do so, allowing for study in syngeneic tumor models. In the case of TPV, it only replicates in humans and monkeys, both naturally and in their cancer tissue(s) [10], which prevents the use of the common syngeneic mouse in vivo models. Therefore, to advance this area of investigation, it was important to develop a new in vivo model that can be used with tropism-limited OVs. To overcome this hurdle, BALB/c nude mice were chosen as xenograft hosts for the MDA-MB-231 human cell line (used previously in our lab [6]), which would then be made ICt via adoptive transfer of splenocytes. This is because the inbred genetic background of these mice allows for immune reconstitution with cells from normal BALB/c donors without inducing host versus graft reactions (specifically immune cells from the donor rejecting the new host), effectively allowing large xenografted tumors to exist in an ICt host for a short period. We have tested this new animal model using human melanoma xenografts previously [11]. In this study, two TPV recombinants known to be successful in treating xenografts in vivo, TPV/Δ66R/mIL-2 and TPV/Δ2L/Δ66R/FliC, were inoculated intratumorally (IT) into BALB/c nude mice bearing human TNBC tumors to test for efficacy and interaction with the host immunity. A timeline representing key points in the experiment is visualized in Figure S1. 

## 2. Materials and Methods

### 2.1. Cells, Viruses, and Reagents

Owl monkey kidney (OMK) cells and human triple-negative cancer (MDA-MB-231) cells were purchased from American Type Culture Collection (ATCC, Rockville, MD, USA) and cultivated at 37 °C with 5% CO_2_. OMK cells were grown in Eagle’s minimum essential medium (EMEM), supplemented with 100 U/mL penicillin, 100 µg/mL streptomycin, 30 mL/L 7.5% NaHCO_3_, and 10% (*v*/*v*) fetal bovine serum (FBS). An identical EMEM solution with 2% (*v*/*v*) FBS (maintenance medium) was used for virus infections and tumor implantation protocols. MDA-MB-231 cells were grown in Leibovitz’s medium (L-15), supplemented with the same components as the EMEM, and had 10% FBS. For recombinant virus replication, OMK cells were infected, as described previously. Expression of the IL-2 and FliC transgenes was previously confirmed [8,9]. TPV/Δ66R/mIL-2 was previously tested for efficacy in an IDt animal model bearing TNBC xenografts in our lab [6]. 

### 2.2. Virus Amplification

Both viruses used were amplified in OMK cells and concentrated 100X using ultracentrifugation (45Ti rotor at 186,000× *g* for 90 min). The concentrated viruses were then titrated in 6-well dishes containing OMK cell monolayers as described previously [12]. Viruses were diluted in sterile phosphate-buffered saline (PBS-A) such that in 100 µL, 5 × 10^6^ plaque-forming units (PFU) would be present for tumor injections. Viruses were stored at −80 °C and, when needed for virotherapy, thawed at 4 °C, sonicated for 8–10 s for uniform suspension, and kept on ice during injections.

### 2.3. Animals

BALB/c athymic nude mice (CAnN.Cg-*Foxn1^nu^*/Crl) and BALB/c normal mice (BALB/cAnNCrl) were purchased between 4 and 5 weeks of age from Charles River and acclimated for 1 week following arrival. All animals were housed, and subsequent treatments were carried out following protocols approved by Western Michigan University’s Institutional Animal Care and Use Committee (IACUC number 19-06-04).

### 2.4. Virotherapy of Human TNBC Xenografts in BALB/c Nude Mice

Tumor xenografts were induced by injection of 5 × 10^6^ MDA-MB-231 cells, mixed 1:1 (*v*/*v*) with Matrigel (Corning Life Sciences, Glendale, AZ, USA), and suspended in 100 µL of sterile Dulbecco’s PBS (DPBS). Both flanks of female BALB/c nude mice (~6 weeks) were injected subcutaneously. Cell viability was tested both prior to injection and post-injection with 0.4% (*w*/*v*) trypan blue in sterile PBS-A to ensure that >90% of the cell population was and remained viable through the procedure. Once tumors became visible, volumes were measured with digital Vernier calipers using the formula ((length × width × height) × (π/6)) in mm^3^ (this has been the standard equation for calculating tumor volume in our lab where π/6 ~ 0.52359 [6,7,8,9,11]). Once tumor volumes reached the range of 120–180 mm^3^, mice were randomly assigned into 1 of 5 possible treatment groups. In virotherapy-treated groups, a single dose of 5 × 10^6^ PFU/100 µL (suspended in sterile DPBS) TPV/Δ66R/mIL-2, TPV/Δ2L/Δ66R/FliC, or wild-type (*wt*) TPV was delivered IT. Tumors from either of the control groups (mock–MC or reconstituted–RC) were injected IT with 100 µL of vehicle. This represented day 0 of treatment. Tumor volumes were then measured independently every other day for a total period of 40 days. The remaining tumor tissue and blood samples were taken and preserved for histopathological analyses. 

### 2.5. Immune Reconstitution

On day 4 post-therapy, all mice not included in the mock control group were reconstituted with whole splenic cells from genetically identical BALB/c normal donor mice. Donor mice were sacrificed via cervical dislocation, and the whole spleen was collected aseptically. The spleen was placed in ice-cold RPMI-1640 + 10% FBS, placed onto a metal sieve, and pressed through using a plastic spatula until the organ disintegrated and individual cells were collected into the medium. The cell mixture was then centrifuged at 1100 rpm (234× *g*) for 5 min at 4 °C. Following centrifugation, cells were resuspended in ice-cold DPBS and centrifuged once more under the same conditions, repeated twice. Cells were then tested for >90% viability as described before and diluted to ~3 × 10^6^ cells/100 µL (roughly 1 × 10^8^ total splenocytes in a mouse spleen). Cells were kept on ice and were injected intraperitoneally (IP) into each mouse. 

### 2.6. Tissue Preparation and Histopathological Analyses

Tumor samples were removed from each treatment mouse at the end of the 40-day period and placed into 10% (*v*/*v*) buffered formalin for 48–72 h. The tissue samples were moved to 60% ethanol for long-term storage until processing occurred. For histological processing, each mass was cut through the center axis, and each cut surface was trimmed and sectioned following standard paraffin embedding. Then, it was stained with hematoxylin and eosin. Two to three sections were cut from each face for examination, and all slides were blind-coded for examination.

### 2.7. Plaque Reduction Assay

Blood taken from sacrificed mice was coagulated for 30 min at 37 °C and then cooled overnight at 4 °C. Following overnight incubation, samples were centrifuged at 12,000 rpm (13,523× *g*) for 10 min at 4 °C in an Eppendorf 5415 R centrifuge. Serum was harvested from these samples and stored at −20 °C. For plaque assays, OMK cells were planted into 6-well dishes, using EMEM + 10% FBS as previously described, and incubated at 37 °C until confluent. When the cells were ready, diluted samples of TPV/Δ2L/Δ66R/FliC were co-incubated at a 1:1 ratio *v*/*v* with 1:10 dilutions of serum in maintenance medium for 3 h at 37 °C. The effective dilution of serum following co-incubation was doubled to 1:20. After co-incubation, samples were added in duplicate to wells, and maintenance medium was added to ensure each well volume was 300 μL. Plates were then placed on a rocker table for 1 h at room temperature for virus adsorption. Following adsorption, 2 mL of overlay medium (2X EMEM + 4% FBS in a 1:1 ratio *v*/*v* with 1% methylcellulose) was added to each well and incubated at 37 °C for 9 days. After the incubation period, medium was aspirated from each well, and 0.5 mL of plaque stain solution (0.4% crystal violet, 37% formaldehyde, and deionized water) was added for 30 min. Each plate was then rinsed under cold water 3 times and allowed to dry. Plaques were then counted and averaged among samples from representatives of treated animals from each group.

### 2.8. Statistical Analyses

Linear mixed models were used to analyze the effects of the treatments on tumor growth curves [13,14]. Analysis was restricted to days 4 through 40 as the virus was administered on day 0, but immune reconstitution did not occur until day 4. Tumor volume on day 4 varied substantially among mice, so the response variable in all analyses was the percent of initial tumor volume (i.e., tumor volume on day 4). The percentage of initial volume was log-transformed to improve model fits.

Tumor growth curves were modeled using second-order orthogonal polynomials. Orthogonal polynomials were used for two main reasons: (1) orthogonal polynomials eliminate potential collinearity between linear and quadratic time terms, and (2) the intercept term in orthogonal polynomial models represents the mean value of the response variable during this study in the reference treatment (i.e., the area under the tumor growth curve; [14]). The MC treatment was set as the reference treatment in the analyses. Initially, four nested models were built. Model 1 contained only orthogonal linear and quadratic time terms. Model 2 included orthogonal linear and quadratic time terms and virus treatments but no interactions between virus treatments and time. Model 3 was identical to Model 2, but it included an interaction between the virus treatments and the linear time term. Finally, Model 4 was the same as Model 3, but it included an interaction between the virus treatments and the quadratic time term. All four models included random effects for each mouse (intercepts and both the linear and quadratic time terms). The models were compared using likelihood ratio tests. All analyses were carried out in R version 3.6.2 (R Core Team 2019) using the lme4 package (version 1.1-26). *p*-values for parameter estimates were obtained using lmerTest (version 3.1-3), and comparisons of treatment means were performed using multcomp (version 1.4-15).

**Figure 1:**
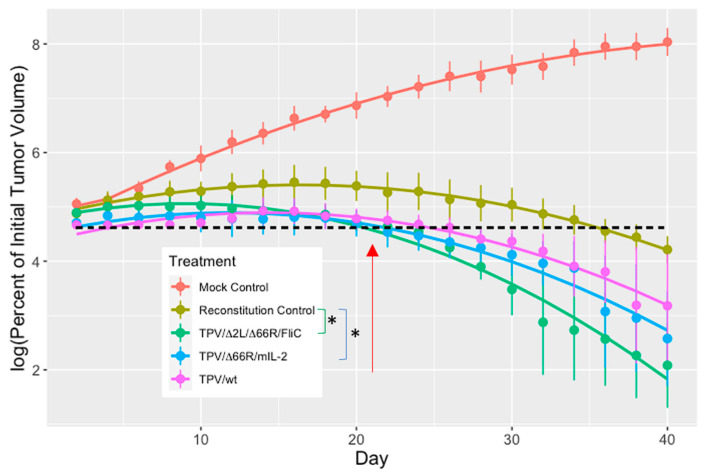
TNBC immuno-deficient and immuno-competent tumor volume analysis. Tumors were induced via subcutaneous injection of 5 × 10^6^ MDA-MB-231 cells, suspended 1:1 (*v*/*v*) in Matrigel, onto the dorsal surface of athymic BALB/c nude mice (just below scapula and above hind leg; each group n = 4 mice). Mice were separated into treatment groups randomly upon development of tumors whose volume reached 120–180 mm^3^ (to simulate more advanced tumors). In the graph above, the vertical axis represents the percent change in tumor volume compared to day 4 volume (log of 100 is 4.60517), and the horizontal axis is time in days. The mock control (MC) and reconstitution control (RC) groups were treated with 100 µL of sterile buffer (DPBS). All mice in TPV-treated groups were injected with 5 × 10^6^ PFU/100 µL of respective TPV. Mice received their treatment dose on day 0, and all immune reconstitutions with whole splenocytes from genetically identical BALB/c donors occurred on day 4 (second data point) with 3 × 10^6^ cells suspended in 100 µL of sterile DPBS. Tumors were measured every other day for 40 days with calipers, and tumor volumes were calculated using the formula ((length × width × height) × (π/6)). The average tumor volume among all mice on each day in mm^3^, for each group, was log-transformed and then compared to the mean initial tumor volume on day 0 for their respective groups to produce the above graph. Solid lines are the predicted percent of initial tumor volume from a linear mixed model. The points are the observed mean percent of initial tumor volume (±1 SE). The overall mean log percent of initial tumor volume did not differ significantly between the RC and *wt*TPV treatments (*p* = 0.08788), but there was a significant difference between the TPV/Δ66R/mIL-2 and TPV/Δ2L/Δ66R/FliC treatments when compared to RC mice (*p* = 0.03890 and *p* = 0.01602, respectively). An analysis of the slope of tumor growth rate on day 21 was significantly more negative in the TPV/Δ2L/Δ66R/FliC treatment than in the RC treatment (*p* = 0.005075). * Indicates a significant difference between groups at each end of the bracket (*p* < 0.05). All analyses were performed using R 3.6.3.

Linear mixed Model 2 did not provide a significantly better fit to the data than Model 1 ($\chi_{3}^{2}$ = 3.397, *p* = 0.3344). However, Model 3 (which included virus treatments and the interaction between virus treatments and the linear time term) received substantially greater support than Model 2 ($\chi_{3}^{2}$ = 33.9792, *p* ≪ 0.0001). Finally, Model 4, which included all possible interactions between virus treatments and the time variables, provided a poorer fit to the data than Model 3 ($\chi_{3}^{2}$ = 2.6894, *p* = 0.4420). This indicates that the general shape of the tumor growth curves did not differ between the virus treatments (tumor growth curves were convex with similar degrees in the shape of the peak in all treatments; see Figure 1). Thus, Model 3 is the basis for all results reported below. Residual plots and normal probability plots were used to examine whether Model 3 satisfied the assumptions of the linear mixed effects model. These plots suggested that the assumptions of the statistical model were not violated.

## 3. Results

### 3.1. Tanapoxvirus-Mediated Virotherapy of Human TNBC Xenografts in Immune Reconstituted Nude Mice Causes Significantly Faster Tumor Regression than Control

MDA-MB-231 cells were implanted subcutaneously into athymic BALB/c-*Foxn1^nu^* mice, supplemented 1:1 (*v*/*v*) with Matrigel at ~6 weeks of age to establish tumors. Tumor xenografts took between 8 and 13 weeks to develop and grow to a volume of 120–180 mm^3^. This higher initial tumor volume threshold was used to simulate the more advanced disease stage seen in many cases of aggressive TNBCs and brought the mice into adult ages during tumor development and subsequent treatment. This is more representative of typical cancer treatment scenarios in humans, where the standard for tumor xenograft experiments is to use juvenile mice, which have different overall immune cell compositions from adult mice. 

Mice were randomly assigned to treatment groups (MC, RC, TPV/Δ66R/mIL-2, TPV/Δ2L/Δ66R/FliC, or *wt*TPV) following the development of tumor volumes into the stated range. Day zero represented when treatment was administered, either 5 × 10^6^ PFU/100 µL of virus or 100 µL of sterile DPBS, intratumorally. Four days later, mice in the RC or virus treatment groups were given 3 × 10^6^ whole spleen cells from genetically identical, healthy BALB/c donor mice, suspended in a 100 µL vehicle, and administered IP. The complete results of tumor virotherapy with TPV recombinants are shown in Figure 1. The overall mean log percent of initial tumor volume was significantly greater in the MC treatment than in all other treatments (RC: *p* = 4.566 × 10^−6^; *wt*TPV: *p* = 1.002 × 10^−10^; TPV/Δ66R/mIL-2: *p* = 7.693 × 10^−12^; TPV/Δ2L/Δ66R/FliC: *p* = 5.748 × 10^−13^). The overall mean log percent of initial tumor volume for all TPV groups was significantly lower than in the RC group (*p* = 0.01163*). The mean log percent of initial tumor volume was also significantly greater in the RC treatment than in the TPV/Δ2L/Δ66R/FliC treatment group (*p* = 0.01602*) and the TPV/Δ66R/mIL-2 treatment group (*p* = 0.03890*). The overall mean log percent of initial tumor volume did not differ between the RC and *wt*TPV treatment groups (*p* = 0.08788). The slope of the tumor growth curve midway through this study (on day 21) was significantly greater in the RC treatment than in the TPV/Δ2L/Δ66R/FliC-treated mice (*p* = 0.005075*) and greater, though not significant for TPV/Δ66R/mIL-2 and *wt*TPV (*p* = 0.155124 and *p* = 0.607054, respectively).

An endpoint analysis was also conducted to determine if there were any significant differences between volumes among treatment groups at the end of this study (day 40). As was expected, the average tumor volume on day 40 was significantly greater in the MC group than in all other groups, there was no significant difference between the RC mice and any TPV-treated animals, and there was no difference between any TPV treatments. Data are presented in Table 1 below.

### 3.2. Plaque Reduction Assay

Following day 40, mice were sacrificed, and blood collected from treated animals was coagulated and centrifuged to separate serum, which could potentially contain antibodies against TPV (if animals were exposed to virotherapy) and/or antigens from the MDA-MB-231 cells. A set of experiments was conducted to determine the presence of anti-TPV antibodies present in serum samples from mice treated with TPV and reconstituted with splenocytes. An additional set of serum samples derived from a previous tumor experiment in this same model, bearing human SK-MEL3 tumors [11], was used in this experiment as well. These mice were treated with the same *wt*TPV and TPV recombinants as in this TNBC study, though one group of mice per treatment was left immune-deficient and received no immune reconstitutions. The non-reconstituted (non-RC) samples were chosen for this experiment. We sought to determine if mature T cells from immune reconstitution with splenocytes were essential to elicit some level of anti-TPV antibodies in these mice or if exposure to viral treatment alone was sufficient.

**Figure 2:**
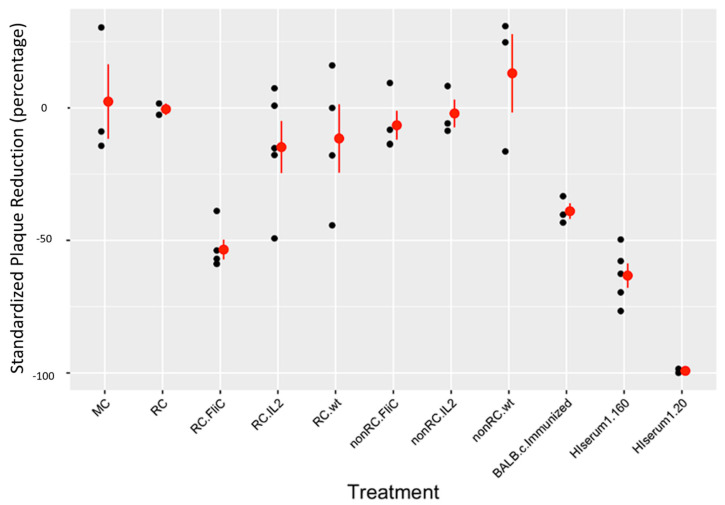
Plaque reduction assay demonstrating anti-viral immunity in treated samples. Blood taken from sacrificed mice was coagulated for 30 min at 37 °C and then cooled overnight at 4 °C. Following overnight incubation, samples were centrifuged at 12,000 rpm (13,523× *g*) for 10 min at 4 °C in an Eppendorf 5415 R centrifuge. Serum was harvested from these samples and stored at −20 °C. For plaque assays, OMK cells were planted into 6-well dishes, using EMEM + 10% FBS as previously described, and incubated at 37 °C until confluent. When the cells were ready, diluted samples of TPV/Δ2L/Δ66R/FliC were co-incubated at a 1:1 ratio *v*/*v* with 1:10 dilutions of serum in maintenance medium for 3 h at 37 °C. The effective dilution of serum following co-incubation was doubled to 1:20. After co-incubation, samples were added in duplicate to wells, and maintenance medium was added to ensure each well volume was 300 μL. Plates were then placed on a rocker table for 1 h at room temperature for virus adsorption. Following adsorption, 2 mL of overlay medium was added to each well and incubated at 37 °C for 9 days. After the incubation period, medium was aspirated from each well, and wells were stained for 30 min with 0.5 mL plaque stain solution. Each plate was then rinsed under cold water 3 times, and plaques were then counted and averaged among samples from representatives of treated animals from each group. Average plaque counts in duplicate were considered in one experimental trial for the purpose of analysis, where plaque values were standardized using the following formula: ((Experimental group average value–TPV control average on trial day)/TPV control average on trial day). Each data point reflects one standardized experimental trial, and the scale on the *y*-axis represents the proportional difference between an experimental group and TPV control average values. Negative values indicate a smaller average plaque count in standardized values than in the TPV control.

During every experimental trial, one set of controls was constant as various test samples were being evaluated: a TPV control (virus only, no serum), a hyper-immune (HI) serum control (virus and either 1:80 dilution of HI serum or 1:10 dilution), and a medium control (no virus, maintenance medium only). This ensured that all trials could be standardized (standardized plaques = (average number of plaques in treatment–average number of plaques in TPV control for trial)/average number of plaques in TPV control for trial) to account for any variation in control plaque numbers from trial to trial. It also ensured no viral contaminants were present in the medium, which could have contributed to overall plaque counts. The results are presented in Figure 2 below. Among the serum groups, 24 planned comparisons were made using one-way ANOVA to determine if there were significant differences between their standardized plaque counts. Of the 24 comparisons, 12 had significant differences, 4 others were nearly significant (*p* > 0.05, but < 0.068), and the remaining 8 comparisons were not significant. These comparisons are summarized in Table 2 below. Negative values in the “value” column indicate the first listed group or combined group’s average standardized plaque number was less than the group or combined group it was being compared to, and positive values indicate greater average standardized plaque number. 

### 3.3. Histopathology 

**Figure 3:**
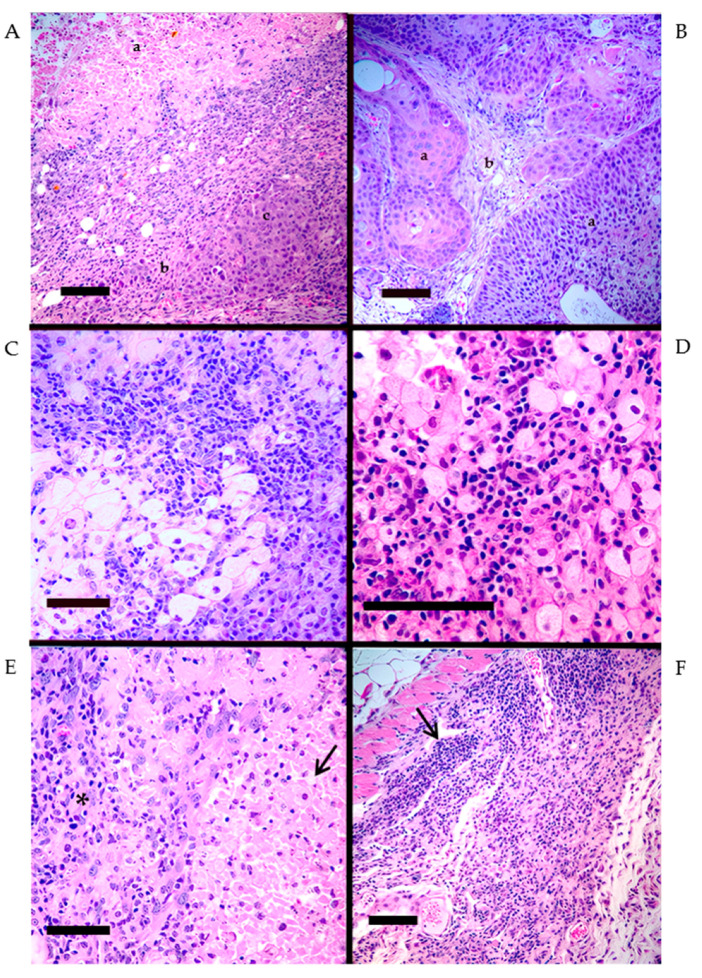
Infiltrating cells. (**A**). Lower power image showing three distinct areas: a. necrosis and hemorrhage (**a**), cellular infiltrate (**b**), and active tumor (**c**). (**B**). Areas of active tumor (**a**) and tissue septa (**b**), all relatively free of infiltrating cells. (**C**). Mononuclear cells with morphology consistent with either lymphocytes and/or macrophages associated with degenerating tumor cells. (**D**). Area of degenerating tumor cells with invading mononuclear cells. (**E**). Area with active cellular infiltrate (*) and necrosis (arrow). (**F**). Tumor tissue replaced with intense accumulations of mononuclear cells (arrow) of lymphocyte morphology. Scale bars are added to each image and represent 100 microns.

**Figure 4:**
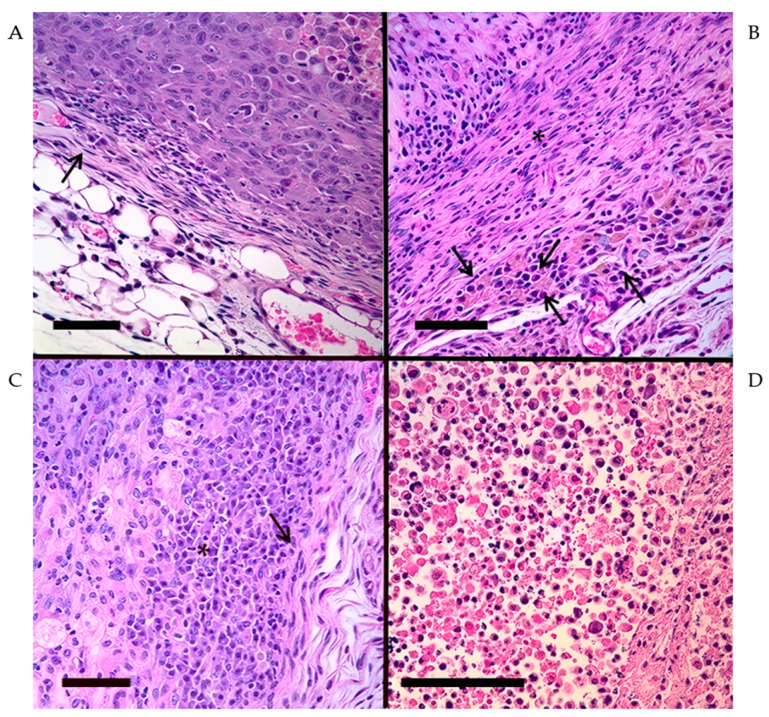
Capsule and necrosis. (**A**). Capsular area with relatively few mononuclear cells and limited fibrosis (arrow). (**B**). Capsular area with extensive fibrosis (*) and a variety of inflammatory cells present (arrows). (**C**). Area of plasma cell accumulation under the capsule (arrow). (**D**). Area of tumor necrosis. Scale bars are added to each image and represent 100 microns.

The overall status of the fixation and staining was of high and of an assessable quality. Tumor material was almost always surrounded by regular host material, allowing clear observation of the peri-tumor areas and assessment of the cellular content of this capsular region. Examples of the various changes seen are shown in Figure 3 and Figure 4. The tumor tissues varied from intact, healthy neoplastic cells to cells that were in various stages of degeneration and necrosis. Inflammatory cells, predominately of small mononuclear morphology, varied considerably between the samples examined in terms of extent and location; cells typical of macrophage–monocyte cell lines were also present on occasion; polymorph cells were only rarely present. Accumulations of small mononuclear cells typical of lymphocyte morphology, and in some cases, morphology typical of plasma cells (Figure 4C), were seen in all areas of the tumor tissues, including the capsule, the fibrous septa, and within the neoplastic cell sheets, the number of cells present at the various locations within the examined tissues ranged from none being present to extensive numbers invading the tissues (Figure 3). 

Sections of each tumor area were examined to address the following aspects: (a) the general status of the tissues; (b) the presence, type, and location of inflammatory cells; (c) the presence of mitotic figures in the tumor cells; (d) the form of the capsular surrounding the tumor tissue; and (e) the degree of tissue degeneration and necrosis present. These characteristics were recorded and scored using the following scale: 0—absent; 1—mild; 2—moderate; 3–extensive; and 4—immense. Then, they were averaged among the samples collected for each group (n = 4) (Table 3).

## 4. Discussion

In a previous study, it was demonstrated that TPV recombinants were able to significantly reduce TNBC cell viability in vitro via direct cell lysis [6], and when tested for efficacy in an IDt mouse model, TPV/Δ66R/mIL-2 was shown to be the most effective at reducing tumor volume when compared to all other virus recombinants tested. It is of note that TPV/Δ2L/Δ66R/FliC was not tested previously in the TNBC model, but rather a TPV recombinant bearing mCCL2 (also known as monocyte chemoattractant protein 1). Based on the results of that study, the next logical progression was to test for efficacy in ICt animals. However, due to TPV’s tropism for only human and monkey cells (both healthy and cancerous), this prevents the use of standard ICt syngeneic tumor models in mice. Other well-studied poxviruses, such as vaccinia virus and myxoma virus, do not share some of the tropism obstacles we find in TPV. Vaccinia virus has been studied extensively in syngeneic mouse models, and a number of recombinant viruses from various labs have been developed for the clinical stage based on those studies. Myxoma virus is a rabbit pathogen and naturally does not infect human or murine hosts; however, it can readily replicate in many tumor cells from mice and humans, even showing efficacy when it replicates poorly [15,16]. TPV’s unique circumstances as an OV led our lab to design a way to create our own model that closely resembles an ICt tumor model to ensure we could progress the development of TPV toward cancer therapy in humans. 

The idea of using immune cells from one animal to replenish the immune system of another is not new. The first experiments testing this concept were carried out in the late 1970s-80s [17,18,19] as a result of work looking into how chemically induced tumors could be rejected in mice and their protection from subsequent challenges [20,21]. However, this idea had not been applied to xenograft models for the purpose of creating an ICt animal from a previously deficient one. The advantage of this model is that it allows for the establishment of xenografted tumors prior to immune reconstitution, allowing the tumor to create an immune suppressive environment that can resist immediate transplant rejection. Tropism-restricted viruses such as TPV can then be studied in an ICt model system without needing to go to monkeys or directly to humans. Since the induction of this study, new humanized models from Jax labs have been developed (hu-PBMC-NSG and hu-PBMC-MHC I/II double knockout, [22]), which potentially offer an alternative route to study xenograft models in humanized mice. Similar to the challenges of the model presented herein, MHC compatibility was a main obstacle to the establishment of grafts in systems with functional T-cell populations. However, administration of IL-2 adversely impacted host survival in the hu-PBMC-MHC I/II mice due to increased induction of graft versus host disease [22], which would pose a potential issue in the use of our TPV/Δ66R/mIL-2 recombinant during cancer studies. 

Because there is such an extensive number of mouse strains with various genetic backgrounds and specific deficiencies, the major obstacle to making this model work was picking a pair of mouse strains that had identical immuno-genetic backgrounds and could offer the largest study window. This was critical as MHC incompatibility can lead to host versus graft disease against the mouse itself, where the transferred immune cells would recognize the mouse as non-self and try to mount an immune response against it. Our choice was through Charles River Laboratories and their BALB/c nude and BALB/c normal mice strains (CAnN.Cg-*Foxn1^nu^*/Crl and BALB/cAnNCrl, respectively). The main disadvantages of this model are that (1) the mice will begin to reject the xenograft once the immune cells fully reconstitute the animal, and the tumor is recognized as containing non-self antigens, and (2) the effects of TPV on off-target tissues cannot be effectively studied in this model when TPV will only replicate in the xenografted tumor. However, we believe that TPV will be selectively replicating in tumor tissues of human origin when viral 66R is deleted. The timing of adoptive transfer and the number of injected splenocytes are critical to make sure the response from the experimental OV(s) is distinguishable from that of the animal’s now fully functional immune system. In the development of this model, it was observed that the use of a whole donor spleen (~1 × 10^8^ cells) led to aggressive rejection of the implanted tumor, where any oncolytic activity would have been difficult to detect through volumetric analysis. Instead, when the spleen was removed and immune cells separated from organ tissue, the cells were collected and diluted to ~3 × 10^6^ total cells in suspension prior to injection. This lower overall number of cells was still sufficient to reconstitute the animal, as approximately 30 percent of the splenocytes in a mouse are T cells. Tumor rejection was observed to begin between days 18 and 20 for the RC group. Graft rejection is known to be a T-cell-mediated reaction, and the nude mice used in our experiment were athymic and defective in T cells. Tumor rejection in the RC group strongly suggests that the T-cell population was replaced and is stable from the adoptive transfers. This effectively suggests that the TPV recombinants and recruited innate immune cells had approximately 2 weeks or so to be the major causes of tumor regression in the early stages of treatment before a T-cell-dependent graft rejection response developed. These characteristics are highly similar to those seen when our lab tested this same mouse model but xenografted melanoma tumor cells instead [11]. 

Our statistical analysis of tumor regression data as a growth curve model may be new to the field of OVs, but this approach is standard elsewhere in statistics. We have used this same approach for tumor modeling in our previous experiments in this animal model [11]. The primary reason that an analysis of this kind was necessary was that a control variable (RC) and all treatment variables (TPV recombinants) had bell-shaped growth curves, whereas the untreated, immune-deficient MC group had an exponential growth curve. This complicates modeling as not all groups are responding similarly to treatment. It was clear during the design of these experiments that there would be a significant difference in tumor volume between RC and MC groups before any involvement of TPV treatments due to inherent tumor transplant rejection mechanisms for all reconstituted groups. Observations from previous unpublished experiments with this model demonstrated that tumors could be resolved completely before the end of the 40 days in the RC group if the entire spleen’s retinue of cells (~3 × 10^8^) from the BALB/c donor was injected into the nude mice as opposed to the diluted amount. Therefore, an analysis of tumor growth and subsequent regression throughout the course of the experiment was a more appropriate approach to determining differences in treatment response between our TPV recombinants and the RC group. In this way, the rate of tumor volume change over time can be used to compare if there are significant effects of virus treatment on the transplant rejection mechanism alone. Separately, an endpoint analysis of tumor volume on day 40 was performed to demonstrate how it is not an effective approach to compare treatment outcomes in this animal model. As the main comparator for our TPV recombinants, the RC group, if given enough time, will eventually reject all transplanted tumors completely. This means the longer the experiment goes on, the less different tumor volumes will be between our control and treatment groups. By basing conclusions of efficacy on this type of endpoint analysis, where the rate at which a tumor volume regresses is ignored, it would support a conclusion that TPV recombinants have no differential effects and are no better than the reconstituted control group in treating TNBC xenografts in this reconstituted BALB/c nude mouse model. However, by instead analyzing treatment effects via rate of change as a log percentage of the initial tumor volume, we demonstrated that both tested TPV recombinants could exert anti-tumor effects significantly more quickly than transplant rejection alone. This fact is further supported by the comparison of the mean percent of initial tumor volume of all TPV treatments to the RC group. In this comparison, the tumor volume was still significantly less for the mean of all TPV groups than the RC group, which suggests that, in general, TPV-mediated treatment is having a significant effect on tumor volume changes observed in this model. 

In this study, two TPV recombinants were chosen for study in the new ICt mouse model, TPV/Δ2L/Δ66R/FliC and TPV/Δ66R/mIL-2, to compare with *wt*TPV and the RC groups. The FliC recombinant was chosen because TNBC cells express TLR5, which is the immune-activating receptor for flagellin-C [23,24]. The immune responses to each of the transgenes are also mediated by different arms of the immune system, primarily innate for FliC and primarily adaptive for IL-2, allowing for a more comprehensive look at which cells might be recruited and factor into tumor regression. Finally, *wt*TPV was chosen as the TPV control group as opposed to TPV/Δ66R because we intended to demonstrate whether the immunostimulatory genes inserted in the other recombinant TPVs were having profound effects on treatment outcome or if *wt* alone was sufficient to cause significant changes in the treated tumors. The results demonstrated that TPV/Δ2L/Δ66R/FliC- and TPV/Δ66R/mIL-2-treated tumors had significantly less average log percent of initial tumor volume over the course of the experiment compared to RC and had greater rates of tumor regression at midpoint than RC. The *wt*TPV group did not demonstrate these significant differences compared to RC, which illustrates the additive effects therapeutic transgenes have on the stimulation of host immunity against the tumor. 

For the examination of pathology for treated tumor sections, a scoring range was made between 0 and 4 to describe to what extent tumor-infiltrating cells had penetrated the capsule or the tumor itself and the status of the tumor from multiple characteristic levels. In the baseline (MC), the tumor was behaving in a classical manner where there was little capsule, mostly healthy and growing tumor, with very limited immune response across the samples (Figure 3B). When observing the RC tumors, the primary driving force behind the immune response is the xenograft transplant rejection (self vs. non-self). This is reflected in changes to the tumor where the capsule is much thicker than the MC samples, there are far higher levels of necrosis and tumor degradation occurring, the tumor has less active tumor cells, and there is even some tumor infiltration by recruited immune cells, though most of them are limited to the capsular area. The major changes seen with TPV/Δ66R/mIL-2-treated samples compared to RC is that the capsule increased in thickness and had more immune cells infiltrating the capsule as well as the tumor body, likely due to the adaptive immune system activation following interleukin-2 signaling. The highest observed levels of immune cell activity occurred within tumor tissues treated with TPV/Δ66R/mIL-2. For TPV/Δ2L/Δ66R/FliC-treated samples, the major difference between this treatment and RC was again capsular thickness and capsular infiltration. Interestingly, this recombinant had generally few cells that infiltrated the tumor body compared to RC (Table 3), but it had the highest rate of tumor volume regression. This is likely due to the more immediate innate-oriented immune response that becomes triggered upon activation of TLR5 with flagellin-C inserted in this virus, which led to tumor section samples where the immune cells may have already left the tumor by the time they were analyzed. If the initial immune response is more quickly recruited to the tumor in this recombinant than in mIL-2 expressing TPV or *wt*TPV, this could explain the lowest mitotic rates observed within the tumor sections of any treated group. It has been shown that the adaptive immune response is also crucial to the generation of antibodies against the flagellin protein, which was not detectable in mice lacking T cells [25]. Furthermore, another independent study previously also showed that TLR5 activation increases IL-2 production and CD4^+^ T-cell proliferation [26]. This would indicate that a FliC expressing OV could be triggering the activation of both arms of the immune response, leading to more complete anti-tumor activity and potentially explaining TPV/Δ2L/Δ66R/FliC-treated mice having the greatest tumor regression rates in our study. It is also of note that the TPV/Δ2L/Δ66R/FliC recombinant includes the deletion of TPV’s TNF-binding protein (2L) to further encourage immune signaling within infected cells [27]. It is likely this also played a key role in the more significant tumor responses made when treated with TPV/Δ2L/Δ66R/FliC than *wt*TPV.

The primary goal of the plaque reduction assays was to determine if the reconstituted mice were capable of producing anti-viral antibodies after treatment with TPV. A unique feature of this experiment was that the mice were not natural hosts for TPV infection; only the transplanted human cancer cells were. Therefore, if the virus was replicating in the tumor, a simulation of infection as a byproduct of treatment would occur. This both allows for an opportunity for the mouse’s immune system to detect TPV and potentially leads to an anti-viral immune response, as well as for the virus to attempt to evade the immune response. Natural TPV infection clears without treatment in humans in approximately 7–9 weeks [28,29,30], partially due to a number of host immunity-augmenting genes carried in its genome, which will only be expressed during an infection, and partially due to its slow rate of infection compared to other poxviruses. Serum samples selected for these experiments were not all from mice harboring TNBC tumors. The serum groups where the mice are considered non-RC but with all of the same TPV recombinants were derived from the previously mentioned experiment in the same mouse model [11]. Due to all other experimental variables being nearly identical, including TPV recombinants used, outside of the tumorigenic cell lines, we believed this addition to be valuable as a set of control samples. If we were correct, immune-deficient and athymic BALB/c nude mice exposed to TPV would not be capable of making neutralizing antibodies, and as seen in Figure 2, that was observed. An important feature of this experiment was the standardization of experimental plaque average values. This was carried out because on each experimental trial, typically, 4 or 5 of the 11 possible groups were tested in duplicate, making direct comparisons between different trials difficult, and TPV control values had minor differences between experiments. This inevitably altered the experimental serum-treated plaque numbers to that trial’s control. Standardizing average plaque numbers in each individual trial allowed for averages to be taken among experimental groups when experiments were repeated at least three times in duplicate, further allowing statistical comparisons to be made between groups. 

In total, there were 24 planned comparisons among the serum groups tested in the plaque reduction assays (Table 2). Of those, the most important comparisons were the following: 4, 8, 12, 15–17, 23, and 24. Comparisons 4, 8, 12, and 15 all demonstrated that the reconstituted serum samples exposed to the same TPV recombinants as the non-reconstituted serum samples had significantly greater plaque reduction values, both when compared individually and when averaged as groups. This suggests that the adoptive transfer of splenocytes allowed for neutralizing antibodies to be made in the treated and reconstituted mice that were not being made in the immune deficient, non-reconstituted counterparts. Comparison 17 demonstrated that immune reconstitution was not triggering a non-specific antibody to be produced, as the RC group showed no differences from the MC group, which also was not exposed to TPV (comparison 23), and there were significantly more plaque reductions in the mice treated with TPV and reconstituted from the RC serum samples. Comparison 24 corroborates this idea, as there were also no differences in plaque reductions for the non-RC serum samples exposed to TPV treatment and the MC group either, meaning both exposure to TPV and adoptive transfer of mature T cells was necessary for the nude mice to make neutralizing antibodies. Finally, comparison 16 was crucial because this was testing to see if there was any difference in plaque reduction between normal BALB/c mice that had been immunized to TPV via four weekly doses of *wt*TPV and the reconstituted BALB/c nude mice treated only once with TPV, but with active replication occurring in the tumors. There was no difference detected between these samples, which suggests that the adoptive transfer allowed for a statistically equivalent anti-viral immune response to be made against TPV in the nude mice as in normal BALB/c mice. Not only are the mice then capable of making neutralizing antibodies following the adoptive transfer of splenocytes, but the immune functionality is being restored to natural levels as well.

Additionally, there are at least three lines of evidence in the data presented here that strongly suggest that the immunological reconstitution of the athymic nude mice was achieved by turning previously IDt mice into ICt ones. These include (a) the reconstitution control, as shown in Figure 1, which clearly demonstrates that the mice that were not injected with TPV but received adoptive transfers of splenocytes began rejecting the tumors while the MC group did not. This can only be achieved via T-cell-mediated immune responses elicited against the tumor. (b) There were lymphocytes observed during tissue section analyses to have infiltrated the tumors of reconstituted animals, both into the capsule and septa, where some of these cells had the morphology typical of plasma cells (Figure 4C). Plasma cells would indicate the induction of antibody production, which typical athymic nude mice are incapable of recruiting in a T-dependent manner. Overall, cellular infiltrates were greater in the RC group than in the MC group, increasing further in virus-treated and reconstituted animals, likely from the addition of transgenes in the recombinant TPVs stimulating immune cell recruitment. (c) Plaque reduction assays using serum acquired from treated mice demonstrate that mice that were immune reconstituted and exposed to TPV treatment had generated some level of neutralizing anti-TPV antibodies, which were not present in mice that were not exposed to TPV nor in mice that remained immune-deficient and were exposed to TPV (Figure 2 and Table 2). Furthermore, comparisons of standardized plaque counts demonstrated no statistical difference between the average of all TPV-treated and reconstituted animals with standardized plaque counts from normal BALB/c mice immunized against TPV. This strongly suggests that T-dependent antigens exposed to the newly intact immune system by TPV were leading to antibody production in the later stages of treatment, which would only occur if mature T cells were present to drive antigen presentation and B cell stimulation. Additionally, there were no differences between the MC and RC group average plaque counts or between MC and the average of all non-RC viral treatment serum groups (harvested from a separate experiment using the same animals, reconstituted in an identical manner). Yet, both comparisons between the standardized, average plaque counts for all reconstituted TPV treatments and their non-RC counterparts were significantly different. This was also the case when comparing reconstituted and TPV-treated groups in comparison to the RC group plaque numbers. This again supports the idea that these mice had successfully been made ICt as only mice that received adoptive transfers of splenocytes and were exposed to TPV had neutralizing antibodies present, which could reduce plaque numbers compared to the TPV control. It could also be hypothesized that anti-MDA-MB-231 antibodies were being generated by these reconstituted mice to help eliminate the tumor xenografts just as they were shown to have generated anti-TPV antibodies. This would, in turn, suggest that anti-tumor immunity was being elicited by activation of the adaptive immune system of the reconstituted nude mice.

### Limitations of This Study

There are a number of limitations to this study that should be addressed. (1) The *wt*TPV group was not included in the pathological analyses. This was due to the *wt*TPV group being tested at a later point in time than the rest of the treatment groups. We have previously shown in non-RC nude mice that *wt*TPV treatment of MDA-MB-231 xenografts did not show significant differences to mock treatment in mitotic index or immune cell invasion into the tumor and detected immune cells were limited to the tumor boundaries [6]. Considering the non-significant tumor response in this reconstituted nude mouse model, we believe it is highly likely that a similar lack of immune cell recruitment occurred during the treatment of these animals, contributing to the lesser rates of tumor regression compared to the recombinant TPVs. However, this remains to be confirmed in this model. (2) As discussed throughout this paper, a feature of this model is that tumor rejection will occur in the RC group regardless of the speed at which the tested OV initiates its own anti-tumor effects. We have shown that the roughly 1 × 10^6^ mature T cells present in the adoptive transfer inoculation, along with the other present immune cells in the donor spleen, were capable of restoring immune function to the nude mice and initiated their own anti-graft responses. We do not yet know at this time whether this number of purified T cells would induce the same effect alone as the non-purified splenocyte population in this study or if there are other essential cells coming from the normal mice that are needed for the restoration of immune function. (3) The number of animals in our study was limited to n = 4. This was primarily due to a tumor take rate of 17/30 for nude mice injected at the inception of this study. None of the 13 mice reinjected with 5 × 10^6^ MDA-MB-231 cells following the initial failure developed tumors on the second attempt. Our initial design was meant for n = 5 in all groups, with the possibility of n = 6 if all mice developed tumors. The *wt*TPV group was added after the conclusion of the initial set of animals and was not included in the 17 mice described. Despite the low number of experimental replicates, the data presented are still clearly significantly different. We have seen similar results with both TPV recombinants in another study in this model system treating melanoma tumors, where n = 6 in those experiments [11]. We believe there could also be a potential role that the high level of NK cells within these BALB/c nude mice are playing in the relatively low tumor take rate we observed in this study. NK cells are well known to have anti-tumor capabilities (reviewed in [31]), and it has been observed that intact NK cell populations can pose obstacles to tumor implantation in other mouse models [32]. Though we did not experience similar issues with low take rates in our melanoma BALB/c nude mouse model [11], it is entirely possible that NK cells were actively inhibiting tumor implantation in this TNBC model. In the absence of an electron microscope in our facility, we were not able to obtain visual evidence for viral factories in examined tumor cells to outline the role of TPV and other relevant cells during treatment. (4) Specific mechanisms of anti-tumor activity for both TPV and any recruited immune cells were not determined in this study. Though we have supporting evidence indicating the crucial role the recombinant TPVs are playing in this model system, both through cell lysis and immune cell recruitment, it is not clear to what extent each component has in the overall tumor regression outcome. We believe it would be necessary to investigate immune cell composition via flow cytometric analysis in future studies at various stages of treatment to better understand major players in the beginning, middle, and end of the treatment window. Though we expect viral lysis and some innate immune activity to be primarily responsible for tumor growth inhibition in the more immediate stages of treatment, we would hypothesize that adaptive immune responses play a major role in the later stages of treatment. This is based on the evidence that these mice are capable of producing neutralizing anti-TPV antibodies and, therefore, likely anti-tumor antibodies as well. It may also be noted that although TPV does not replicate in mice, the potential antigens in TPV are fully capable of inducing both B- and T-cell-mediated immune responses in mice.

## 5. Conclusions

The main objective of this study was to determine the potential efficacy of tested TPV recombinants against TNBC in an ICt model system. The results showed that TPV/Δ2L/Δ66R/FliC and TPV/Δ66R/mIL-2 both demonstrated significant differences in the rate of regression of tumor volume over the course of the experiment compared to RC animals, whereas *wt*TPV did not. Endpoint analysis also showed that there were no significant differences on day 40 between RC and any TPV-treated groups. This is an important observation for our model because, generally, OV studies use endpoint analyses to determine significant anti-tumor activity, as untreated tumors will always grow exponentially. In the case of our model, a known feature is that tumor regression via transplant rejection will occur naturally after the adoptive transfer of the splenocytes that include the missing mature T cells for these nude mice. Through trial and error, we believe that this model has been adjusted to allow a window of time for an OV to exert its own anti-tumor pressure, which can be measurable and statistically analyzed for significant effects. Having a control group reject a tumor by itself could easily be considered a limitation of this model, but at this point in time, there are no other options available to study tropism-limited OVs in an ICt model when viral tropism does not include mice or other standard model systems. For TPV, there are no available monkey tumor cell lines that can be purchased to do such experiments, which necessitated the development of this immune-reconstituted nude mouse model to get as close as possible to that of a syngeneic mouse tumor model. The results reported support the continued study of TPV’s interactions with the immune system in this model to further understand the roles of recruited immune cells and their effects on the tumor volume changes we observed.

## Figures and Tables

**Table 1:** Day 40 endpoint analysis of tumor volume.

Group Comparison	Value	Df	Sum of Square	F Value	*p* Value of F
MC–RC	4.0736	1	36.876	26.2356	0.0007168 ***
MC–TPV/Δ2L/Δ66R/FliC	5.6503	1	70.946	50.4754	2.496 × 10^−5^ ***
MC–TPV/Δ66R/mIL-2	5.3210	1	62.919	44.7641	4.669 × 10^−5^ ***
MC–*w*tTPV	5.0151	1	55.891	39.7645	8.365 × 10^−5^ ***
RC–TPV/Δ2L/Δ66R/FliC	1.5767	1	4.972	3.5374	0.4699678
RC–TPV/Δ66R/mIL-2	1.2475	1	3.112	2.2143	0.7809198
RC–*wt*TPV	0.9415	1	1.773	1.2613	1.0
TPV/Δ2L/Δ66R/FliC–TPV/Δ66R/mIL-2	−0.3293	1	0.217	0.1543	1.0
TPV/Δ2L/Δ66R/FliC–*wt*TPV	−0.6352	1	0.807	0.5741	1.0
TPV/Δ66R/mIL-2–*wt*TPV	−0.3059	1	0.187	0.1332	1.0

***: *p* < 0.0001.

**Table 2:** Planned one-way ANOVA comparisons among anti-viral immunity serum groups.

Group Comparison	Value	df	Sum of Square	F Value	*p* Value of F
1. RC.FliC–RC.*wt*	−0.41931	1	0.39071	14.1020	0.0008067 ***
2. RC.FliC–RC.IL2	−0.38677	1	0.37399	13.4983	0.0009997 ***
3. RC.FliC–(RC.*wt* + RC.IL2)/2	−0.40304	1	0.51982	18.7618	0.0001718 ***
4. RC.FliC–nonRC.FliC	−0.46926	1	0.48935	17.6623	0.0002435 ***
5. RC.FliC–BALB/c Immunized	−0.14500	1	0.03942	1.4229	0.2429329 (NS)
6. RC.FliC–RC	−0.53014	1	0.40149	14.4912	0.0007038 ***
7. RC.IL2–(RC.FliC + RC.*wt*)/2	0.17712	1	0.10039	3.6233	0.0673007 .
8. RC.IL2–nonRC.IL2	−0.12718	1	0.03033	1.0947	0.3043867 (NS)
9. RC.IL2–BALB/c Immunized	0.24177	1	0.10960	3.9558	0.0565623 .
10. RC.IL2–RC	−0.14336	1	0.02936	1.0598	0.3120829 (NS)
11. RC.*wt*–(RC.FliC + RC.*wt*)/2	0.22592	1	0.14583	5.2635	0.0294852 *
12. RC.*wt*–nonRC.*wt*	−0.24614	1	0.10386	3.7485	0.0630098 .
13. RC.*wt*–BALB/c Immunized	0.27431	1	0.12899	4.6556	0.0396797 *
14. RC.*wt*–RC	−0.11083	1	0.01638	0.5911	0.4484284 (NS)
15. ((RC.FliC + RC.IL2 + RC.*wt*)/3)–((nonRC.FliC + nonRC.IL2 + nonRC.*wt*)/3)	−0.28086	1	0.45316	16.3558	0.0003732 ***
16. ((RC.FliC + RC.IL2 + RC.*wt*)/3)–BALB/c Immunized	0.12369	1	0.03772	1.3616	0.2531020 (NS)
17. ((RC.FliC + RC.IL2 + RC.*wt*)/3)–RC	−0.26144	1	0.11945	4.3114	0.0471535 *
18. HIserum1.160–BALB/c Immunized	−0.24301	1	0.11073	3.9965	0.0553841 .
19. HIserum1.160–((RC.FliC + RC.IL2 + RC.*wt*)/3)	−0.36670	1	0.49398	17.8292	0.0002308 ***
20. HIserum1.160–RC.FliC	−0.09801	1	0.02401	0.8668	0.3598122 (NS)
21. HIserum1.160–RC.IL2	−0.48478	1	0.58754	21.2061	8.161e-05 ***
22. HIserum1.160–RC.*wt*	−0.51732	1	0.59471	21.4649	7.561e-05 ***
23. MC–RC	0.02860	1	0.00098	0.0354	0.8520566 (NS)
24. MC–((nonRC.FliC + nonRC.IL2 + nonRC.*wt*)/3)	0.00918	1	0.00019	0.0070	0.9339436 (NS)

*** > 0.001, * > 0.05, “.” > 0.1.

**Table 3:** Tumor section analysis **.

Sample	Capsule Thickness	Capsule-Infiltrating Cells	Tumor Necrosis and Degeneration	Tumor Mitotic Rate	Tumor-Infiltrating Cells	Tumor Cell Uniformity
MC	1	1.25	2.5	2.5	0.75	2.75
RC	2.5	3	3.5	0.5	1.75	1.75
TPV/Δ66R/mIL-2	3.75	3.75	3.5	0.25	3	1.75
TPV/Δ2L/Δ66R/FliC *	3	3.67	3.33	0	0.67	1

* TPV/Δ2L/Δ66R/FliC had 1 sample where a complete response was made, and no tumor was present at sacrifice; averages were taken with n = 3 instead of n = 4 for that group. ** All given values are averages of the scores given to each tumor analyzed for each mouse per group.

## Data Availability

The data presented in this study are available on request from the first author as they are included in his Ph.D. thesis.

## Supplementary Materials

The following supporting information can be downloaded at https://www.mdpi.com/article/10.3390/pathogens13050402/s1. Figure S1: Triple-negative breast cancer xenograft tumor model experimental timeline.
