# Supplementary material for: Oncolytic Tanapoxvirus Recombinants Expressing Flagellin C or Mouse Interleukin-2 Are Capable of Regressing Human Triple-Negative Breast Cancer Xenografts in Immuno-Competent BALB/c Nude Mice
Source: Pathogens. 2024 May 13;13(5):402. doi: 10.3390/pathogens13050402 (PMC11124456; doi:10.3390/pathogens13050402)
Supplement: Supplementary file 1 [file pathogens-13-00402-s001.zip › pathogens-2917046-supplementary.pdf]

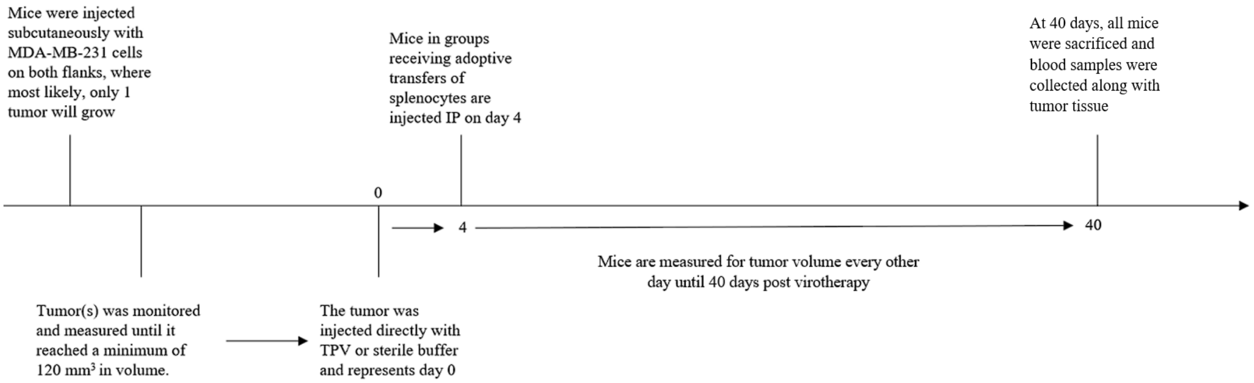

Figure S1. Triple-negative breast cancer xenograft tumor model experimental timeline. Tumors were induced via subcutaneous injection of 5×10^6 MDA-MB-231 cells, suspended 1:1 (v/v) in Matrigel, onto the dorsal surface of athymic BALB/c nude mice (just below scapula and above hind leg; each group n = 4 mice). Mice were separated into treatment groups randomly upon development of tumors whose volume reached 120-180 mm^3 (to simulate more advanced tumors). The mock control (MC) and reconstitution control (RC) groups were treated with 100 μL of sterile buffer (DPBS). All mice in TPV treated groups were injected with 5×10^6 PFU/100 μL of respective TPV. Mice received their treatment dose on day 0 and all immune reconstitutions with whole splenocytes from genetically identical BALB/c donors occurred on day 4 (second data point) with 3×10^6 cells suspended in 100 μL of sterile DPBS. Tumors were measured every other day for 40 days with calipers and tumor volumes calculated using the formula ((length × width x height) × (π/6)). At 40 days, all mice were sacrificed and blood samples were collected along with tumor tissue.
